# CRITICAL EVALUATION OF LONG-TERM RESULTS OF MALIGNANT HEPATIC TUMORS TREATED BY MEANS CURATIVE LAPAROSCOPIC HEPATECTOMY

**DOI:** 10.1590/0102-6720201700030010

**Published:** 2017

**Authors:** Sergio Renato PAIS-COSTA, Sergio Luiz Melo ARAÚJO, Olímpia Alves Teixeira LIMA, Sandro José MARTINS

**Affiliations:** 1 Hospital Santa Lucia; 2 Hospital Brasília, Brasília, DF, Brazil

**Keywords:** Laparoscopy, Hepatectomy, Liver neoplasms/surgery, Neoplastic metastasis

## Abstract

**Background:**

Laparoscopic hepatectomy has presented great importance for treating malignant hepatic lesions.

**Aim:**

To evaluate its impact in relation to overall survival or disease free of the patients operated due different hepatic malignant tumors.

**Methods:**

Thirty-four laparoscopic hepatectomies were performed in 31 patients with malignant neoplasm. Patients were distributed as: Group 1 - colorectal metastases (n=14); Group 2 - hepatocellular carcinoma (n=8); and Group 3 - non-colorectal metastases and intrahepatic cholangiocarcinoma (n=9). The conversion rate, morbidity, mortality and tumor recurrence were also evaluated.

**Results:**

Conversion to open surgery was 6%; morbidity 22%; postoperative mortality 3%. There was tumor recurrence in 11 cases. Medians of overall survival and disease free survival were respectively 60 and 46 m; however, there was no difference among studied groups (p>0,05).

**Conclusion:**

Long-term outcomes of laparoscopic hepatectomy for treating hepatic malignant tumors are satisfactory. There is no statistical difference in relation of both overall and disease free survival among different groups of hepatic neoplasms.

## RESUMO

**Racional:**

A hepatectomia laparoscópica tem apresentado grande importância no tratamento das lesões hepáticas malignas.

**Objetivo:**

Avaliar o impacto dela realizada por uma única equipe em relação à sobrevida global e tempo livre de doença nos diferentes tumores malignos hepáticos.

**Métodos:**

Foram realizadas 34 hepatectomias laparoscópicas em 31 pacientes com neoplasia maligna. Os doentes foram distribuídos em: Grupo 1 - metástases colorretais (n=14); Grupo 2 - carcinoma hepatocelular (n=8) e Grupo 3 - metástases não-colorretais e colangiocarcinoma intra-hepático (n=9). As curvas de sobrevida e sobrevida livre de doença foram estimadas. Foram avaliadas também a taxa de conversão, morbidade, mortalidade e recorrência tumoral.

**Resultados:**

A taxa de conversão foi de 6%; a morbidade de 22%; a mortalidade pós-operatória de 3%; recorrência tumoral em 11 casos. As medianas de sobrevida global e de sobrevida livre de doença foram respectivamente de 60 e 46 m, contudo não houve diferença entre os grupos estudados (p>0,05).

**Conclusão:**

Os resultados em longo prazo da hepatectomia laparoscópica para o tratamento de tumores malignos hepáticos são satisfatórios. Não houve diferença estatisticamente significante quanto às sobrevidas global e livre de doença nos diferentes grupos de neoplasia tratada.

## INTRODUCTION

The first totally laparoscopic standard hepatectomy was performed at the beginning of the 1990s. This procedure then quickly became the main method of choice for treating different forms of hepatic neoplasia. Although at first laparoscopic hepatectomy (LH) was indicated mostly for benign hepatic neoplasia, new studies showed that this approach was also safe and effective for treating both primary and metastatic malignant tumors^2^ ^,^ ^3^ ^,^ ^7^ ^,^ ^10^ ^,^ ^29^. LH has now become the method of choice for treating malignant hepatic neoplasia in many centers worldwide^1^ ^,^ ^5^ ^-^ ^7^ ^,^ ^11^ ^,^ ^12^ ^,^ ^13^ ^,^ ^14^ ^,^ ^15^ ^,^ ^17^ ^,^ ^20^ ^,^ ^23^ ^-^ ^25^ ^,^ ^27^. 

Although there is a lack of randomized clinical trials with large samples in the literature, studies with lower levels of scientific evidence have demonstrated some advantages of this method in comparison with open hepatectomy (OH)^7^. The main findings have been: better analgesic effect, lower use of opiates, less bleeding, lower rate of postoperative complications (both hepato-specific and pulmonary), shorter hospitalization time and better cosmetic results^1^ ^,^ ^5^ ^-^ ^7^ ^,^ ^11^ ^,^ ^12^ ^,^ ^13^ ^,^ ^14^ ^,^ ^15^ ^,^ ^17^ ^,^ ^20^ ^,^ ^23^ ^-^ ^25^ ^,^ ^27^. Analogous results have been observed regarding treatment of malignant forms of neoplasia specifically, compared with OH results^1^ ^,^ ^13^. Overall and disease-free survival, as well as recurrence rates, are similar between LH and OH. A minimally invasive approach may result in better preservation of the remaining hepatic parenchyma, without compromising oncological principles^1^ ^,^ ^2^ ^,^ ^3^ ^,^ ^10^ ^,^ ^13^ ^,^ ^19^ ^,^ ^24^. Also in Brazil, previous studies from the present group and also studies from other researchers have shown that LH was safe and effective for treating malignant hepatic lesions^14^ ^,^ ^16^ ^,^ ^21^.

 The objective of the present study was to compare the outcomes from different groups of malignant hepatic tumors that were operated through laparoscopy. 

## METHODS

Between June 2007 and January 2016, 31 patients with malignant hepatic neoplasia underwent 34 LH procedures. The patients were distributed into the following groups: Group 1 - colorectal metastases (n=14); Group 2 - hepatocellular carcinoma (n=8); and Group 3 - non-colorectal metastases and intrahepatic cholangiocarcinoma (n=9). The cases of non-colorectal metastases consisted of the following: renal carcinoma (n=1), non-functioning neuroendocrine pancreatic tumor (n=3), small-intestine adenocarcinoma (n=1), ovarian germ tumor (n=1) and invasive ductal carcinoma (n1). 

The mean age of the participants was 53.4 years, and the median age was 52 years (range: 34-78). Regarding gender, there were 18 men and 13 women. The size of lesions ranged from 2-8 cm, with a mean of 3.8 cm. Among the patients with metastases, nine presented more than one lesion. In all patients, the primary origin of the disease was colorectal, although in four patients the distribution was bilateral. All were asymptomatic regarding the hepatic tumor and the findings were incidental.

 The indications for LH were: lesions smaller than 6 cm (up to three or four lesions), preferentially in one lobe or sector (especially the left lobe or right posterior sector), far from large vessels (hepatic vein, portal vein, vena cava or hepatic artery) and far from central positions. Specifically, in the case of hepatocarcinoma, the guidelines standardized by the BLCL (“Barcelona Liver Cancer Clinic”) were adopted. Therefore, for small localized tumors, in Child A or non-cirrhotic patients, without portal hypertension and in good condition, the always-anatomical LH option was used. In turn, for patients with non-colorectal metastases, criteria previously published by one of the authors of the present study were adopted^8^. Thus, HL was performed in cases of a single metachronic metastasis (in general at least one year of disease-free survival after radical treatment of the primary tumor without evidence of local recurrence) or a small number of lesions (<3), generally restricted to one lobe or extrahepatic disease-free segment, for which the primary tumor was considered to present a good prognostic. On the other hand, there was a larger range of criteria for LH in colorectal metastases, in accordance with the tendency in the literature. Specifically, in cases of intrahepatic cholangiocarcinoma, hilar hepatic lymphadenectomy was performed in addition to LH, as recommended by the TNM-UICC.

 All LH were defined in accordance with the terminology of the International Hepato-Pancreato-Biliary Association (IHPBA), derived from the Coinaud classification, following the Brisbane 2000 nomenclature. Subsequently, larger hepatectomy was defined as resection of three or more hepatic segments. When a biliary fistula was present, it was defined and classified using the IHPBA classification, in accordance with the International Study of Liver Surgery (ISGLS), 2011. Ultrasonography, computed tomography and magnetic resonance imaging of the abdomen were performed on all the patients. For the last 10 patients, both PET-CT and resonance with hepatobiliary-specific contrast (Primavist) were also performed. Serum tumor markers, such as carcinoembryonic antigen, alpha-fetoprotein, Ca 15.3 and Ca 19.9, were assessed in all cases. For neuroendocrine tumors, chromogranin A and neuron enolase were administered, respectively.

 For LH, three standardized techniques that had previously been reported in detail by the present authors^9^ ^,^ ^21^ ^,^ ^22^ were used. The techniques were, respectively: intrahepatic Glissonian access^28^ - “posterior approach”, using the technique proposed by Machado et al.^16^; extra-Glissonian access -“anterior approach”, using the technique proposed by Takasaki^26^; and the classical Coinaud technique with individualized dissection, isolation and ligature of hilar structures. Pneumoperitoneum of 12-14 mmHg and a 30° laparoscope were generally used. Three to six punctures were performed according to the case, and the lead surgeon performed the operation through patients’ legs (the authors suggest that the studies cited should be read for further technical details). 

 Surgical specimens were removed intact inside an Endobag plastic container or gloves. The preferential incision for removing specimens was an infra-umbilical transversal incision of Pfannenstiel or Maylard type. However, in some cases, median mini-laparotomy or a small direct subcostal incision was used in patients who presented this type of incision previously from other operations. In the liver bed, a hemostatic Surgicel was used and fibrin glue (Eviscel) was then used, when available, to finish the hemostasis. Finally, thin tubular drains were placed in the liver bed, in larger hepatectomies. The drains were subsequently removed when the drainage rate was below 50 ml per day, concomitantly with a serous or serous-hemorrhagic appearance, and without bilious appearance, on two consecutive days. In uncertain cases, bilirubin assays were performed on the drained liquid. If the quantity of bilirubin drained was three times higher than the quantity of serum, a biliary fistula was diagnosed and classified as A, B or C, in accordance with the IGHFB classification. Subsequently, the biliary fistula was treated in a case-dependent manner and according to the availability of the type of treatment in the hospital.

 For all the cases, overall survival was measured from the day of the surgery to the patient’s death, including death due to cancer or other causes, or until the last follow-up day, understood as the final follow-up or death. The disease-free period, defined as the period until the first diagnosed recurrence of cancer after hepatic resection, confirmed by means of biopsy or imaging, was also calculated. Specifically, for stratified statistical analysis, groups of patients were formed, as follows: Group 1 - comprising colorectal metastases (n=14); Group 2 - comprising hepatocellular carcinoma (n=8); and Group 3 - comprising non-colorectal metastases and intrahepatic cholangiocarcinoma (n=9).

### Statistical analysis

 Survival and disease-free survival curves were estimated using the Kaplan-Meier method. The Breslow test (generalized Wilcoxon) was used to make comparisons between groups. P-values < 0.05 were considered statistically significant. The SPSS 17.0 software (SPSS, USA) was used for this analysis 

## RESULTS

The characteristics of all 31 patients are shown in Table 1. The preoperative radiological evaluation showed solid tumors in all lesions except in one case of solid and cystic metastasis due to an ovarian germ tumor. For all the patients of this study, the diagnosis was made from typical radiological imaging findings. PET scans were also performed in the last 10 cases. Tumor markers were also assessed. However, the final diagnosis was only confirmed through histopathological examinations on the surgical specimens. 

**TABLE 1:** Characteristics of patients, lesions, operations performed and immediate surgical results

Case	Gender	Age	Etiology	n	Size (cm)	Type of hepatectomy	Previous surgery	Morbidity
1	m	63	HCC	1	3	RPLS + RALS	-	-
2	m	61	MCR	2	3,5	LLH	Open right colectomy	Pneumonia
3	f	32	NCRM(small intestine)	2	3	LLLS	Open enterectomy	-
4	f	43	NCRM(kidney)	3	3	RPLS	Open right nephrectomy	-
5	m	63	HHC	1	3	RPLS	-	-
6	f	43	CRM	3	3	RLH	Open retosigmoidectomy	-
7	f	54	CRM	2	3	LLLS	Open right colectomy	-
8	m	50	CRM	3	3	LLLS	Laparoscopic low anterior rectum resection	-
9	f	53	CRM	1	4	RPLS	Open left colectomy	-
10	m	65	CRM	3	4,5	Two-stage hepatectomy SIIIL+portal embolization RLH	Laparoscopic retosigmoidectomy	-
11	m	34	CRM	1	2,7	SVIL	Open retosigmoidectomy	-
12	f	78	CRM	1	2,3	RPSL	Open retosigmoidectomy	Pneumonia
13	f	71	CRM	3	3,5	Two-stage hepatectomy LLLS RPLS	Laparoscopic retosigmoidectomy	-
14	m	58	HCC	1	6	LLLS	-	-
15	f	43	CHC	1	4	LLH	-	-
16	m	71	IHC	1	8	RLH	-	sirs + shock + death
17	f	38	NCRM(Ovary)	1	6	LLLS + LHT simultaneous	-	-
18	m	54	NCRM (NET)	4	2	LLLS + RPLS - Enucleation of simultaneous pancreas-tail NET	-	-
19	f	73	CRM	2	3,7	RLH	Open right colectomy	-
20	m	54	NCRM (NET)	4	2,1	LLLS+ SIVBL	Distal open pancreatectomy	-
21	m	72	HCC	1	2	RPLS *	-	-
22	m	73	IHC	1	5	LLLS	Laparoscopic cholecystectomy	-
23	m	71	HCC	1	7	LLLS	-	-
24	f	56	CRM	2	3	LRH	Right flank loop ileostomy right colectomyby means open laparotomy	Open conversion biliary fistula- degree a + eventration + entero-cutaneous fistula
25	m	60	NCRM (NET)	4	2,7	LLLS + SVIL	Dlp	-
26	m	64	CRM	3	3,5	RPLS	Laparoscopic retosigmoidectomy	Biliary fistula- degree a
27	m	73	HCC	1	4	RPSL	Open cholecystectomy and gastrectomy	Intra-operatory bleeding + paralytic ileum
28	f	38	NCRM (breast)	2	3	LLLS	-	-
29	f	54	CRM	6	6	LLH + SVIII+SVII **	Open Hartman procedure	- Conversion intra-operatory bleeding -biliary fistula degree b
30	m	72	HCC	1	3	RPLS*	-	-
31	m	49	CRM	5	5	RPSL+SIIIL+SIVBL	-	-

HCC=hepatocellular carcinoma; CRM=colorectal metastasis; NCRM=non-colorectal metastasis; IHC=intrahepatic cholangiocarcinoma; NET=neuroendocrine tumor; ASA=American Society of Anesthesiologists; RLH=right laparoscopic hepatectomy; LLH=left laparoscopic hepatectomy; RPLS=right posterior laparoscopic sectionectomy; RALS=right anterior laparoscopic sectionectomy; LLLS=left lateral laparoscopic sectionectomy; DLP=distal laparoscopic pancreatectomy; LHT=laparoscopic hysterectomy;*=use of the Habib 4X radiofrequency device; ** atypical resection.

The hepatectomy was completely laparoscopic in 29 patients. There were two conversions in this sample (6%) and there was no intraoperative mortality in this study. The distribution of the surgical techniques used is shown in Table 1. More than one hepatic resection was performed in eight patients. In three of them, resections were performed in two stages (cases 1, 10 and 13; Table 1). In the remaining cases, multiple hepatic resections were performed concomitantly in a single intervention (cases 18, 20, 25, 29 and 31; Table 1). In two patients, in addition to hepatectomy, another non-hepatic operation was performed at the same time, also via laparoscopy (cases 17 and 18; Table 1) and there was one case of enucleation of a non-functioning neuroendocrine tumor of the pancreatic tail. 

The volume of intraoperative bleeding ranged from 0-1000 ml with a median of 352 ml. The duration of the operation ranged from 70-323 min with a median of 173 min. Eight patients needed blood transfusion, and two of them underwent conversion of the procedure to laparotomy. Both of these cases were converted because of firm adherences between the liver and intestinal loops. Both of these patients underwent previous emergency open surgery due to an obstructive colorectal tumor and both presented enterostomy. One of these patients presented an ileostomy exactly in the right hypochondrium, which was moved to the left hypochondrium. Seven patients (22%) presented at least one postoperative complication, and four of them (13%) were considered hepato-specific. The complications presented were as follows: intraoperative bleeding (n=2), biliary fistula (n=3), evisceration with enterocutaneous fistula (n=1) and SIRS with postoperative shock (n=1). The patients who underwent conversion presented at least two postoperative complications (Table 1).

 The postoperative mortality rate (up to the 30^th^ day) was 3%. The only postoperative death was of a 71-year-old obese elderly patient (case 16) who presented diabetes and arterial hypertension. He presented an 8 cm intrahepatic cholangiocarcinoma located between segments V and VI. Right laparoscopic hepatectomy was performed without intraoperative intercurrences, but on the 3^rd^ postoperative day he suddenly presented SIRS with refractory postoperative hemodynamic shock without hematimetric repercussion, followed by death. 

 The duration of hospitalization ranged from two to 63 days with a median of six. Oral diet was reintroduced after 12-24 h in the cases of minor resections, and after 2-3 days in the cases of major resections. Twenty patients needed small doses of common painkillers, such as dipyrone or acetaminophen, for short periods of time. The time taken for the patients to return to daily activities ranged from 7-120 days, with a median of 16 days. 

 The histopathological evaluation showed that 94.5% of the patients who underwent surgery (n=29) presented neoplasia-free margins, while two cases in the present sample were microscopically compromised (cases 1 and 13). In one of these cases (case 13), the margins were microscopically compromised in the second operative specimen. This patient went back to systemic chemotherapy, but died 19 months after the first intervention. 

 In this sample, 25 patients underwent chemotherapy at some moment during their treatment. The follow-up times ranged from 5-80 months, with a median of 42 months. In total, there were 11 recurrences (35%); the organ most often compromised was the liver itself, with seven cases (63%), followed, respectively, by the lungs, bones and pelvis, with two cases each (18%), and the peritoneum, with one case (9%). In four of these cases (36%), the recurrences were multiple (more than one organ; Table 2). The median overall survival for all patients was 60 months (50% in five years).

**TABLE 2:** Long-term recurrence and survival results

Etiology	Overall length of survival (months)	Disease-free survival (months)	Recurrence site	Status
HCC	74	74	-	Alive
CRM	60	47	Pelvis	Dead - cancer
NCRM (small intestine)	13	8	Peritoneum + bones	Dead-cancer
NCRM (kidney)	35	30	Bones and lungs	Dead-cancer
HCC	38	33	Liver	Dead-cancer
CRM	62	46	Pelvis	Dead-cancer
CRM	47	28	Liver	Dead-cancer
CRM	61	61	-	Live
CRM	39	39	-	Live
CRM	46	46	-	Live
CRM	43	29	Liver + lungs	Dead-cancer
CRM	33	33	-	Alive
CRM	29	22	Liver + bones	Dead-cancer
HCC	7	7	-	Mortal- bleeding from esophageal varices
HCC	18	18	-	Alive
IHC	13	8	Liver	Dead-cancer
NCRM(Ovary)	34	34	-	Alive
NCRM(NET)	52	32	Liver	Alive
CRM	28	-	-	Alive
CRM	26	-	-	Alive
HCC	30	18	Liver	Dead-cancer
IHC	18	-	Pelvis	Alive
HCC	15	-	Peritoneum + bones	Alive
CRM	13	-	Bones and lungs	Alive
NCRM (NET)	11	-	Liver	Alive
CRM	9	-	Pelvis	Alive
HCC	7	-	Liver	Alive
NCRM (breast)	7	-	-	Alive
CRM	6	-	-	Alive
HCC	6	-	-	Alive
CRM	5	-	Liver + lungs	Alive

CRM=colorectal metastasis; NCRM=non-colorectal metastasis; NET=neuroendocrine tumor; HCC=hepatocellular carcinoma; IHC=intrahepatic cholangiocarcinoma

However, the length of survival differed between the groups, and was highest in Group 1 (colorectal metastasis), i.e. 60 months, vs. 38 months in Group 2 (hepatocellular carcinoma) and 35 months in Group 3 (non-colorectal metastasis and intrahepatic cholangiocarcinoma), respectively, as shown in Table 3. However, no statistically significant difference was observed between these results (p=0.167).

**TABLE 3:** Median de tumor-specific survival (until death due to cancer) in months*

Group	Median			
	Estimate	Standard-error	95 % Confidence interval	
			Lower limit	Upper limit
1 (CRM)	60,000	14,175	32,218	87,782
2 (HCC)	38,000	12,728	31,053	80,947
3 (NCRM-IHC)	35,000	16,192	3,265	66,735
Overall	60,000	11,461	37,536	82,464

*=the estimate will be limited to the longer survival time, if censored; CRM=colorectal metastases, HCC=hepatocellular carcinoma; NCRM=non-colorectal metastases; IHC=intrahepatic cholangiocarcinoma

**FIGURE 1:**
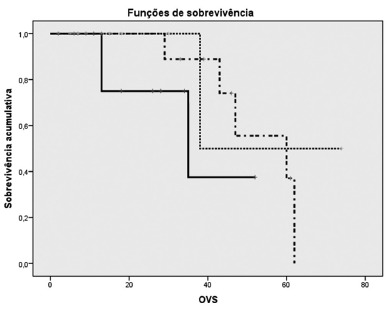
Kaplan-Meier curve for overall survival per group (in months)

**Figure 2:**
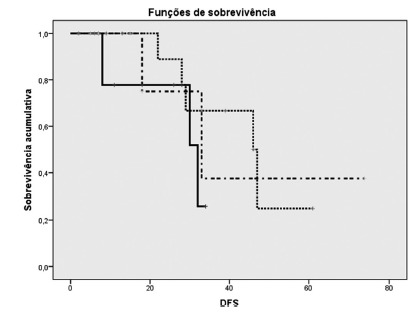
Kaplan-Meier curve for disease-free survival per group (in months)

The median disease-free survival for all the patients was 46 months. Group 1 (colorectal metastasis) also presented a higher median disease-free survival of 46 months, vs. 33 in Group 2 (hepatocellular carcinoma) and 32 in Group 3 (non-colorectal metastasis and intrahepatic cholangiocarcinoma), respectively, as shown in Table 4. However, no statistically significant difference was observed between these groups (p=0.407). Both the overall and the disease-free survival curves (in months) of the different study groups are shown in Figures 1 and 2.

**TABLE 4:** Median disease-free survival (until time of relapse) in months

Group	Median			
	Estimate	Standard-error	95 % Confidence interval	
			Lower limit	Upper limit
1 (CRM)	46,000	10,029	12,344	51,656
2 (HCC)	33,000	11,456	10,545	55,455
3 (NCRM-IHC)	32,000	10,029	12,344	51,656
Overall	46,000	11,311	23,830	68,170

*=the estimate will be limited to the longer survival time, if censored; CRM=colorectal metastases, HCC=hepatocellular carcinoma; NCRM=non-colorectal metastases; IHC=intrahepatic cholangiocarcinoma.

Group 1 (CRM); Group 2 (HCC); Group 3 (NCRM-IHC); CRM=colorectal metastases, HCC=hepatocellular carcinoma; NCRM=non-colorectal metastases; IHC=intrahepatic cholangiocarcinoma

Group 1 (CRM); Group 2 (HCC); Group 3 (NCRM-IHC); CRM=colorectal metastases, HCC=hepatocellular carcinoma; NCRM=non-colorectal metastases; IHC=intrahepatic cholangiocarcinoma

## DISCUSSION

Hepatic surgery is still one of the most challenging and technical surgery procedures currently performed. It depends on wide clinical-surgical knowledge and vast experience. Even when performed by highly-experienced surgeons in reference centers with large samples of patients, hepatectomies present considerable morbidity and mortality. Since the first laparoscopic hepatectomies were performed during the 1990s, expansion of their indications and better results have been observed, in parallel with better knowledge of the technique. Thus, this procedure has become the main choice for treating hepatic neoplasia, and especially for neoplasia in so-called “laparoscopic hepatic segments”, i.e. in anterolateral positions^3^. With improvements during the past decade, patient samples with higher numbers of larger resections located in anatomical positions that are difficult to access, such as posterosuperior or medial segments, have presented good results. Hence, this access route is an interesting alternative in comparison with laparotomy^29^.

 Although LH is considered to be a complex surgical procedure, left lateral laparoscopic sectionectomy is still considered by various specialists to be the easiest hepatic anatomical resection. It has also been the main resection performed in several samples of patients, as can be seen in the initial study by the present authors. Even with expansion of this series to the current sampled of patients, which was more than three times the size of the initial sample and also had a proportionally higher number of more complex and larger resections, left lateral laparoscopic sectionectomy accounted for nearly one third of all resections performed by the present team (31%). This change has also been observed in the literature: the largest proportion of the resections at the beginning of the LH era consisted of sectionectomy or bisectionectomy, especially of segments II, III, IVB, V and VI7. However, in the present study, as well as in the literature, there was a proportional increase in larger resections and right resections, such that, for instance, right posterior laparoscopic sectionectomy accounted for approximately 28% of the surgical cases in the present sample.

 The general advantages of LH follow those that have already been widely mentioned regarding other abdominal procedures^6^ ^,^ ^7^ ^,^ ^9^, with fewer hepato-specific postoperative complications than in HA^1^ ^-^ ^7^ ^,^ ^10^. The conversion rate was 6%, which was similar to what was observed by Cai et al.^4^, Cipriani et al.^6^ and Etorre et al.^11^; lower than what was reported by Montalti et al.^18^ (15.8%) and Nachmany et al.^19^ (11.9%) in cases of malignant neoplasia; and slightly higher than what was reported by Machado et al.^16^ and Lacerda et al.^14^ in mixed samples (both benign and malignant neoplasia). However, the fact that 13 of the 31 patients of the present study (more than 30%) had previously undergone laparotomy, prior to LH, needs to be highlighted. In addition, two patients underwent larger complex resections. As reported by other authors, the main cause of conversion was the intraoperative incidental finding of firm adherences, which limit the progression of the videolaparoscopic technique. In our viewpoint, perhaps those converted cases were no well selected for laparoscopic approach, so after this critical analysis, the position of present team has been no indicate it on patients wich underwent previous open colorectal surgey due complicated tumors with estomy. 

 The overall number of postoperative complications (22%; n=7) was similar to what can be seen in the literature, which has ranged from 15.7 to 23.8% among oncological patients^6^ ^,^ ^18^ ^,^ ^19^ ^,^ ^23^. In the present sample of patients, this rate can be explained by the large number of patients (30%) who presented previous laparotomies. In parallel, the number of hepato-specific complications (13%), which also accounted for all the major complications (n=4), was similar to what was found by Cipriani et al ^,7^ . These complications occurred in 75% of the cases in which patients underwent an abusive chemotherapy scheme, using both irinotecan and oxaliplatin. These drugs are known to be hepatotoxic, given that they lead to steatohepatitis (“yellow-liver”) and sinusoidal hypertension (“blue-liver”), respectively. Use of these drugs may be associated with higher frequency of hepato-specific complications, such as biliary fistulas, and also with perioperative bleeding^28^. However, the re-operation rate was low (3%). 

The mortality of this series was 3%, close to the results described in the literature^1^ ^,^ ^14^ ^-^ ^16^. The only death in the present study was of a patient who presented a large intrahepatic cholangiocarcinoma and underwent right laparoscopic hepatectomy. This patient presented diabetes mellitus type 2, hypertension and obesity, and was an elderly person. These risk factors associated with major hepatectomy, especially on the right side, with resection of 70% of the hepatic mass, have been correlated in the literature with higher postoperative mortality. 

 In the present study, the histopathological evaluation showed that 94.5% of patients who underwent surgery presented a surgical index of R0 (neoplasia-free microscopic margins). This result was similar to what was previously observed by Hilal et al.^12^ and Nachmay et al.^19^.

 In total, there were 11 cases of recurrence (35%), and the organ most often compromised was the liver, with seven cases (63%). Recurrence in the liver is the site most commonly reported in the literature, in relation to both colorectal and non-colorectal metastases^12^.

 A few studies with long-term results in relation to LH for treating malignant primary or metastatic hepatic tumors have been reported. Although so far no meta-analysis on randomized clinical trials has been performed, and there are no large trials comparing LH with OH with this objective, case-control or meta-analysis studies have indicated that LH is not inferior to OH and that, moreover, it presents the clear advantages mentioned earlier. 

 In the present study, an overall median 60-month (5-years) survival rate among all the patients of 50% was observed, which was similar to the findings from other similar laparoscopy studies. The disease-free median survival among all the patients was 46 months. Group 1 (colorectal metastasis) also presented the highest disease-free median survival (46 months), and this was longer than what was found by Montalti et al.^18^. Group 2 (hepatocellular carcinoma) presented disease-free median survival of 33 months. This was also observed in the literature, probably associated with the high rate of recurrence of this neoplasia^11^ ^,^ ^17^. Finally, in Group 3 (non-colorectal metastasis and intrahepatic cholangiocarcinoma), the disease-free median survival was 32 months, which was close to what was observed in Group 2, but lower than what was observed in Group 1. The explanation for this may be that Group 3 was composed by tumors with high rates of recurrence and worst final prognosis^1^. However, perhaps because of the small casuistic of the present study, no statistically significant difference was observed between the three groups regarding disease-free survival (p=0.407).

 Although there is a lack of studies in the literature with higher levels of evidence, it was seen in the present study that both long-term overall and long-term disease-free survival can be attained through use of technique reported here. Thus, this technique did not compromise the oncological treatment in relation to the final prognosis. Although there was no difference in this regard among the different groups treated exclusively using LH, it cannot be categorically stated from these results that such differences really so not exist. Therefore, studies with larger samples of patients or meta-analyses should be conducted in order to answer this question.

## CONCLUSION

The long-term results from LH for treating malignant hepatic tumors were satisfactory. No statistically significant difference was observed in relation to overall and disease-free survival between the groups of different malignant hepatic tumors treated using LH.
